# The Effect of Tissue Preparation and Donor Age on Striatal Graft Morphology in the Mouse

**DOI:** 10.1177/0963689717744788

**Published:** 2018-04-11

**Authors:** David J. Harrison, Victoria H. Roberton, Ngoc-Nga Vinh, Simon P. Brooks, Stephen B. Dunnett, Anne E. Rosser

**Affiliations:** 1 Brain Repair Group, School of Biosciences, Cardiff University, Cardiff, United Kingdom

**Keywords:** cell transplantation, Huntington's disease, primary embryonic tissue, striatal grafts, mouse models, medium spiny neurons

## Abstract

Huntington's disease (HD) is a progressive neurodegenerative disease in which striatal medium spiny neurons (MSNs) are lost. Neuronal replacement therapies aim to replace MSNs through striatal transplantation of donor MSN progenitors, which successfully improve HD-like deficits in rat HD models and have provided functional improvement in patients. Transplants in mouse models of HD are more variable and have lower cell survival than equivalent rat grafts, yet mice constitute the majority of transgenic HD models. Improving the quality and consistency of mouse transplants would open up access to this wider range of rodent models and facilitate research to increase understanding of graft mechanisms, which is essential to progress transplantation as a therapy for HD. Here we determined how donor age, cell preparation, and donor/host strain choice influenced the quality of primary embryonic grafts in quinolinic acid lesion mouse models of HD. Both a within-strain (W-S) and a between-strain (B-S) donor/host paradigm were used to compare transplants of donor tissues derived from mice at embryonic day E12 and E14 prepared either as dissociated suspensions or as minimally manipulated tissue pieces (TP). Good graft survival was observed, although graft volume and cellular composition were highly variable. The effect of cell preparation on grafts differed significantly depending on donor age, with E14 cell suspensions yielding larger grafts compared to TP. Conversely, TP were more effective when derived from E12 donor tissue. A W-S model produced larger grafts with greater MSN content, and while high levels of activated microglia were observed across all groups, a greater number was found in B-S transplants. In summary, we show that the effect of tissue preparation on graft morphology is contingent on the age of donor tissue used. The presence of microglial activation in all groups highlights the host immune response as an important consideration in mouse transplantation.

## Introduction

Huntington's disease (HD) is an inherited neurodegenerative disease caused by an expanded polyglutamate cytosine-adenine-guanine (CAG) repeat in the Huntingtin gene on chromosome 4^1^. The resultant mutant Huntingtin protein leads to progressive neuronal dysfunction and loss, with medium spiny neurons (MSNs) primarily affected in the early stages of the disease^2^. HD is a debilitating disease causing a broad range of physical and mental deficits, and currently there is no disease-modifying treatment.

The relatively targeted nature of the primary neuronal loss in HD makes it an ideal candidate for cell replacement therapy. Primary neuronal progenitors derived from the whole ganglionic eminence (WGE; from which the striatum develops) and transplanted directly into the quinolinic acid (QA)-lesioned striatum develop into MSNs, integrate into the parenchyma, and form functional connections with host neural circuitry in rats^3,4^. A number of clinical trials confirm that transplantation of fetal WGE in HD is safe, and there is preliminary evidence that it can improve some disease symptoms in people with HD.^5–8^ However, more work is required. First, tests must be conducted to determine whether fetal transplantation can reliably improve function in people with HD, and second it should be determined whether the efficacy and consistency of this approach can be improved, before its development as a potential treatment. Moreover, optimizing the protocols for achieving successful embryonic WGE grafts will solve many issues that are also relevant to pluripotent stem cell–derived grafts, which are currently being developed as a more sustainable source of donor cells.

Protocols have been optimized and are well established for rat-to-rat striatal transplants, with extensive preclinical literature showing consistent large and functional grafts from embryonic day (E)14 to E16 WGEs^9,10^. Rat protocols have been refined over many years, with donor age and tissue preparation identified as critical factors affecting graft survival, morphology, and function of rat-to-rat striatal transplants^11–13^; however, these factors have not been systematically investigated in mice. It is evident throughout the literature,^14–18^ and from experience within this lab, that the direct translation of these protocols to mice results in considerable graft variability. Graft survival is lower and surviving grafts are smaller and contain less striatal-like tissue compared to rat striatal grafts. This suggests that either there are unrecognized differences between rat and mouse host models and the way in which they interact with transplanted tissue or species differences in the way the transplanted tissue develops following transplantation, or both.

Typically, immunosuppressive treatment is not required when transplanting rat cells into the rat brain, even using outbred stocks, with good graft integration and functional recovery^19,20^. It has therefore been assumed that transplants in mice would also not require immunosuppression in the supposed “immune-privileged” brain. However, as the immunological response to transplanted tissue is likely to be critical for graft survival, we have considered the host response to transplantation (including the disparity in immunological background between donor and host and the preparation of transplanted cells) as a potential factor in survival of neural mouse grafts.

Establishing a reliable protocol in the mouse is essential to use the array of well-characterized genetic HD mouse models for cell transplantation, as well as a wide range of transgenics that could be used to contribute to a better understanding of graft survival integration and functional mechanisms. The present study examined whether modifications to current standard transplant protocols could produce more reliable and effective striatal grafts in mouse models of HD, both within and between strains. The effects of donor tissue age and cell preparation were assessed by characterizing the cell content of striatal grafts of mouse primary embryonic tissue and analyzing the activated microglial response. The QA lesion model of HD was used, as it is the most widely used and well-validated model to date for preclinical studies and provides a reliable starting point for later translation to genetic models of HD.

## Materials and Methods

This experiment was subject to project, personal, and facilities licenses and local ethical review in accordance with the United Kingdom Animals (Scientific Procedures) Act 1986 as amended.

### Subjects

Young adult male C57BL6/J (*N* = 32) and CD1 (*N* = 32) mice (20 to 30 g, Harlan, Bicester, UK) were housed in pairs under standard conditions in a 12:12-light/dark cycle. Temperature and humidity were maintained at 21 ± 2 °C and 60% ± 1%, respectively. Food and water were available *ad libitum*.

### QA Lesion Surgery

Mice received unilateral QA (P6320-4; Sigma-Aldrich, Gillingham, UK) lesions to the right striatum, with 2 mice of each strain retained as intact controls. Fresh 0.09 M QA solution was prepared each day in 0.1 M phosphate buffer (10010-056; Thermo Fisher, Loughborough, UK). All animals were anesthetized in an induction chamber using 4% isoflurane gas in oxygen, the head shaved, and a subcutaneous (sc) injection of meloxicam 2.5 mg/kg (Metacam, Boehringer Ingelheim, Germany) given as pain relief prior to surgery. Mice were transferred to a stereotaxic frame and maintained on 1.5% to 2% isoflurane in a mixture of oxygen and nitrous oxide (2:1). The skull was exposed and a small hole drilled at the following stereotaxic coordinates: anterior-posterior (AP) +0.8 mm and medial-lateral (ML) −2.0 mm from bregma. A 30-gauge stainless steel cannula attached to a 10 µL microvolume syringe (2035; SGE Analytical Sciences, Thermo Fisher) driven by a mechanical pump was used to inject 0.75 µL of 0.09 M QA at dorso-ventral (DV) −3.0 mm below dura. The QA was infused over 6 min and the cannula left in position for an additional 3 min to prevent back flow of solution. The cannula was removed and the incision closed using 5-0 Vicryl dissolvable sutures (W9915; Ethicon, Livingston, UK). 0.5ml of 0.9% glucose saline (FKE1323; Baxter, Newbury, UK) was administered sc during surgery to reduce dehydration and a 7.5 mg/kg intramuscular injection of diazepam (Hameln Pharmaceuticals Ltd, Gloucester, UK) was given post-anesthesia prevent seizures. Mice were placed into a warm recovery chamber for 2 to 3 h until completely awake and returned to their home cages for 10 d. The general health of mice was monitored daily and mice were fed a wet mash of standard food in their cages for at least 3-d post surgery. In the week following lesion surgery, 7 C57BL6/J mice became unwell, necessitating hand-feeding of wet mash via a syringe daily until weight was regained. In addition, 4 pairs of C57BL6/J mice were separated due to fighting. Animals that did not fully recover from illness or fighting were removed from the study (*n* = 8).

### Donor Tissue

Two transplant paradigms were used incorporating common strain combinations studied within the lab: a within-strain (W-S) model with CD1 tissue transplanted into CD1 hosts and a between-strain (B-S) model with Chrm4-EGFP-CD1 tissue transplanted into C57BL/6J hosts. The CD1 mouse is used as a standard transplantation model for assessing graft survival and composition, chosen primarily for their large litter sizes. The C57BL/6J/Chrm4-EGFP-CD1 model is used to investigate the functional efficacy of transplants, as C57BL/6J mice are particularly adept at performing behavioral tasks and are the background strain for many of the genetically modified HD mouse models. The bacterial artificial chromosome (BAC) Chrm4-EGFP-CD1 mice express green fluorescent protein (GFP) attached to M4 receptors in a subset of MSNs^19^, allowing easy identification of donor-derived MSNs.

Time-mated CD1 and Chrm4-EGFP-CD1 mice from an in-house colony (originally purchased from Harlan, and MMRRC, Farmington, CT, USA, respectively) were sacrificed by cervical dislocation at E12 or E14, and the embryos dissected into Dulbecco’s modified Eagle’s medium: nutrient mixture F-12 (DMEM/F12; 12634-028; Thermo Fisher). Using a dissecting microscope in a laminar flow hood, the brains were removed and, following a longitudinal cut in the medial cortex, the whole (medial and lateral) striatal primordium was identified on the floor of the lateral ventricle and removed via a horizontal cut as described^21^. Four transplant preparations were made for each donor strain: (1) E12 cell suspension (CS), (2) E12 tissue pieces (TP), (3) E14 CS, and (4) E14 TP. Transplantation surgery was spread across multiple days with fresh suspensions made each morning for each group.

### Transplantation Surgery

Approximately 10-d postlesion mice were randomly assigned to experimental groups with 20 C57BL6/J and 27 CD1 mice receiving primary tissue transplants (*n* = 4 to 7 per group, see Table 1). In addition, a group of mice from each strain were retained as lesion-only controls (C57BL6/J, *n* = 2; CD1, *n* = 3). Surgery was conducted using the same anesthetic regime described for lesions; however, no diazepam was administered post-transplantation. Cell preparations were injected at the lesion coordinates via the same burr hole, −3.2 and −2.8 mm below dura.

**Table 1.** Summary of Survival Rates and Untransformed Data for Surviving Grafts.

Host Strains	Groups	Number of Surviving Grafts	Graft Volume (×10^6^ µm^3^)	Number of NeuN^+^ Cells (×10^3^)	P-zone Volume (×10^6^ µm^3^)	Number of DARPP-32^+^ Cells (×10^3^)	Percentage of DARPP-32^+^ Patches (%)
C57BL6/J	E12 CS	4 of 5 (80%)	110.3 ± 52.4	11.1 ± 5.1	61.2 ± 42.4	1.4 ± 0.7	51.0
C57BL6/J	E12 TP	5 of 5 (100%)	301.6 ± 88.5	28.4 ± 8.0	180.6 ± 67.7	2.5 ± 0.8	55.5
C57BL6/J	E14 CS	4 of 4 (100%)	188.9 ± 32.7	12.9 ± 4.1	127.6 ± 5.7	2.4 ± 0.4	74.4
C57BL6/J	E14 TP	2 of 6 (33%)	97.0 ± 16.3	6.8 ± 0.5	44.7 ± 3.7	0.8 ± 0.5	48.1
CD1	E12 CS	5 of 6 (83%)	226.0 ± 52.9	11.1 ± 2.4	91.1 ± 31.1	2.1 ± 0.5	34.0
CD1	E12 TP	6 of 7 (86%)	335.6 ± 46.5	16.4 ± 2.8	237.5 ± 66.4	4.2 ± 0.7	68.4
CD1	E14 CS	6 of 7 (86%)	194.0 ± 21.6	9.6 ± 1.4	84.3 ± 16.9	2.3 ± 0.4	44.3
CD1	E14 TP	3 of 7 (43%)	146.9 ± 36.8	6.6 ± 1.6	119.0 ± 25.6	2.5 ± 0.3	89.9

*Note*: Untransformed data presented ±standard error of the mean. High graft survival rates were seen in most groups with the exception of those derived from E14 TP. Large differences in graft volume and cell numbers were observed within group. CS = single-cell preparation; TP = tissue piece.

### Single-cell Preparations

CS preparations consisted of pooled E12 WGEs (Chrm4-EGFP-CD1, *n* = 26; CD1 *n* = 24) or E14 WGEs (Chrm4-EGFP-CD1, *n* = 22; CD1, *n* = 26) for each strain. Tissue was incubated at 37 °C for 10 min in 0.1% bovine trypsin (25300-054; Thermo Fisher) + 0.05% deoxyribonuclease (DNase) (D4527; Sigma-Aldrich) in DMEM/F12 solution, before adding 0.01% bovine trypsin inhibitor (T6522-250MG; Sigma-Aldrich) for an additional 5 min, and washing with direct addition of DMEM/F12 followed by centrifugation for 3 min at 1,000 rpm. Cells were resuspended in DMEM/F12 and triturated using a Gilson pipette with a 200 µL tip to mechanically dissociate into a single CS. Cell number and viability were determined with trypan blue (T8154 20ML; 0.4% trypan blue solution, Sigma-Aldrich, UK) exclusion using a hemocytometer, confirming all suspensions had >90% viability. Cells were concentrated at 250,000 cells/µL for transplantation in DMEM/F12. 1 µL of suspension was injected at each depth using a 10 µL microvolume syringe (2035; SGE Analytical Sciences, Thermo Fisher), depositing approximately 500,000 cells in total into the lesioned striatum over 2 min (1 µL/min), with the syringe left in situ for an additional 3 min to allow diffusion and reduce backflow. All suspensions were kept in the dark at room temperature.

### Tissue Piece Preparations

For TP preparations, no cell counts could be conducted directly from nondissociated tissue, therefore WGE units equating to approximately 500,000 cells (the number of cells transplanted in the CS groups) were transplanted. Cell counts calculated from the CS dissections showed this to equal approximately a pair of WGEs for E12 tissue and a single WGE for E14 (see Table 2). Separate preparations were made for each individual surgery, with WGEs treated with bovine trypsin, DNase, and trypsin inhibitor as described above. However, after gentle washing, tissue was transferred directly into ∼4 µL DMEM/F12 for transplantation, with no trituration, therefore minimizing mechanical manipulation to maintain integrity of the TPs. TP preparations were injected as above, at a rate of 1 µL/min over 4 min (2 min at each depth). Mice were monitored daily until full recovery.

**Table 2.** Summary of Transplanted Cell Numbers and Associated Proportion of WGE Used in Each Group.

Donor Strains	Embryonic Age	Preparation	Cells per WGE	Proportion WGE Transplanted	Number Cells Transplanted
Chrm4-EGFP-CD1	E12	CS	180,769	2.77	500,000
Chrm4-EGFP-CD1	E12	TP	180,769	2.00	361,538
Chrm4-EGFP-CD1	E14	CS	577,273	0.87	500,000
Chrm4-EGFP-CD1	E14	TP	577,273	1.00	577,273
CD1	E12	CS	357,143	1.40	500,000
CD1	E12	TP	357,143	2.00	714,286
CD1	E14	CS	1,041,667	0.48	500,000
CD1	E14	TP	1,041,667	1.00	1,041,667

*Note*: The number of cells per WGE was estimated based of the mean cell counts of the CS preparations. The number of WGEs used in the TP preparations was adjusted based on the mean number of cells in the WGE for each particular donor strain and age with the aim of transplanting a similar number of cells in each group. Since it was only possible to use whole WGE units in the nondissociated TP preparations, the number of cells transplanted could not be exactly matched but was kept as close to 500,000 as possible. Subsequently, the proportion of WGE transplanted was used to transform the data to account for the differences in proliferative potential of the cells transplanted. CS = single-cell preparation; TP = tissue piece; WGE = whole ganglionic eminence.

### Perfusion and Immunohistochemistry

At 12 wk after transplantation surgery, mice were perfused and the brains were processed for histological analysis of the grafts. Animals received a terminal intraperitoneal injection of sodium pentobarbital (Euthatal, Merial Animal Research, Woking, UK) and were transcardially perfused using phosphate buffered saline (PBS, pH 7.3) followed by 150 mL of 4% paraformaldehyde solution (PFA, pH 7.3; 10131580; Fisher Scientific, Loughborough, Lutterworth, UK) over 4 min. Brains were removed, postfixed in 4% PFA for 4 h, and transferred to 25% sucrose solution in PBS for at least 48 h. Brains were cut at 40 µm on a freezing microtome, and sections stored in antifreeze—5.45 g disodium-hydrogen-orthophosphate (28029.26; VWR, UK), 1.57 g sodium-dihydrogen-orthophosphate (28013.264; VWR), 300 mL ethylene glycol (102466-2.5L; Sigma-Aldrich), and 300 mL glycerol (G7893-2L; Sigma-Aldrich) in 400 mL dH_2_O—at −20 °C until immunohistochemical analysis. The 1:12 series were incubated at room temperature as free-floating sections with primary antibodies for neuronal nuclei (NeuN) (MAB377; 1:2,000; Millipore, Watford, UK), ionized calcium-binding adapter molecule 1 (Iba1) (019 19741; 1:8,000; Wako, Chuo-ku, Japan), parvalbumin (P3088; 1:4,000; Sigma-Aldrich) or anti-GFP (AB11122; 1:1,000; Invitrogen, Loughborough, UK), and streptavidin–biotin reaction (PK-6100; Dako, Glostrup, Denmark), then stained using 3,3′-diaminobenzidine (DAB, D5637-1G; Sigma-Aldrich). Parvalbumin series were double-stained with dopamine- and cAMP-regulated phosphoprotein antibody (DARPP-32) (1:30,000; the kind gift of Professor H. C. Hemmings, Cornell University^22^) and Vector SG kit (SK-4700; Dako). Sections were mounted onto gelatinized slides and left to air-dry overnight before being dehydrated and cover-slipped with distyrene plasticizer and xylene (DPX) mounting medium (12658646; Fisher Scientific).

### In Vitro Primary Cultures

Time-mated CD1 dams were sacrificed at E12 or E14 (*n* = 3 per group), and WGEs were dissected as described previously^21^. Tissue from each litter was pooled to prepare 3 separate suspensions for each embryonic age, as described above. Cells were resuspended in neuronal differentiation media—DMEM/F12 + 1% FCS (10270-106; Thermo Fisher, Waltham, MA, USA) + 2× B27 (17504-044; Thermo Fisher, Waltham, MA, USA) and plated on poly-l-lysine treated coverslips at 100,000 cells per well. CS of 30 µL was left for approximately 1 h before flooding with 500 µL of differentiation media and incubated at 37 °C in humidified 5% CO_2_ and 95% atmospheric air. A complete media change was performed after 3 d in culture using the same media described above. After 24 h and 7 d *in vitro*, 12 wells of each suspension were fixed with 4% PFA and stored at 4 °C until immunocytochemical staining.

### Immunocytochemistry

Cells were quenched in 100% ethanol for 2 min, washed 3 times in PBS, and then blocked with PBS + 0.3% Triton X-100 (PBST; X100-500ML; Sigma-Aldrich) + 1% BSA (A3059; Sigma-Aldrich) + 1% serum at RT for 1 h. Cells were then incubated at 4 °C overnight with the following pairs of primary antibodies in PBS + PBST + 1% BSA + 1% horse serum (16050-122; Thermo Fisher, USA): neuronal marker βIII-tubulin (T2200; 1:500; Sigma-Aldrich) and astrocyte marker glial fibrillary acidic protein antibody (GFAP AB32010; 1:500; Abcam, Cambridge, UK) or early MSN marker forkhead box P1 (FoxP1 AB16645; 1:500; Abcam) and COUP-TF-interacting protein 2 (CTIP2) AB18465; 1:500; Abcam, UK). Cells were washed with PBST before incubating for 2 h in the dark at RT with the following fluorescent secondary antibodies in PBS (1:200): Alexa594 (A11037; Thermo Fisher, UK) for βIII-tubulin and CTIP2 and Alexa488 (A11034; Thermo Fisher, UK) for GFAP and FoxP1. After washing with PBS, a Hoechst (23000-1000; 1:10,000; Fisher Scientific) counterstain was applied for 5 min. Cells were washed again in PBS and coverslips mounted onto microscope slides with aqueous mountant (PBS: glycerol; G7893-2L; Sigma-Aldrich, 1:1) and stored in the dark at 4 °C. Five regions per coverslip were counted, and the mean count from each suspension recorded.

### Analysis of Grafts and Statistics

**Fig. 1.**
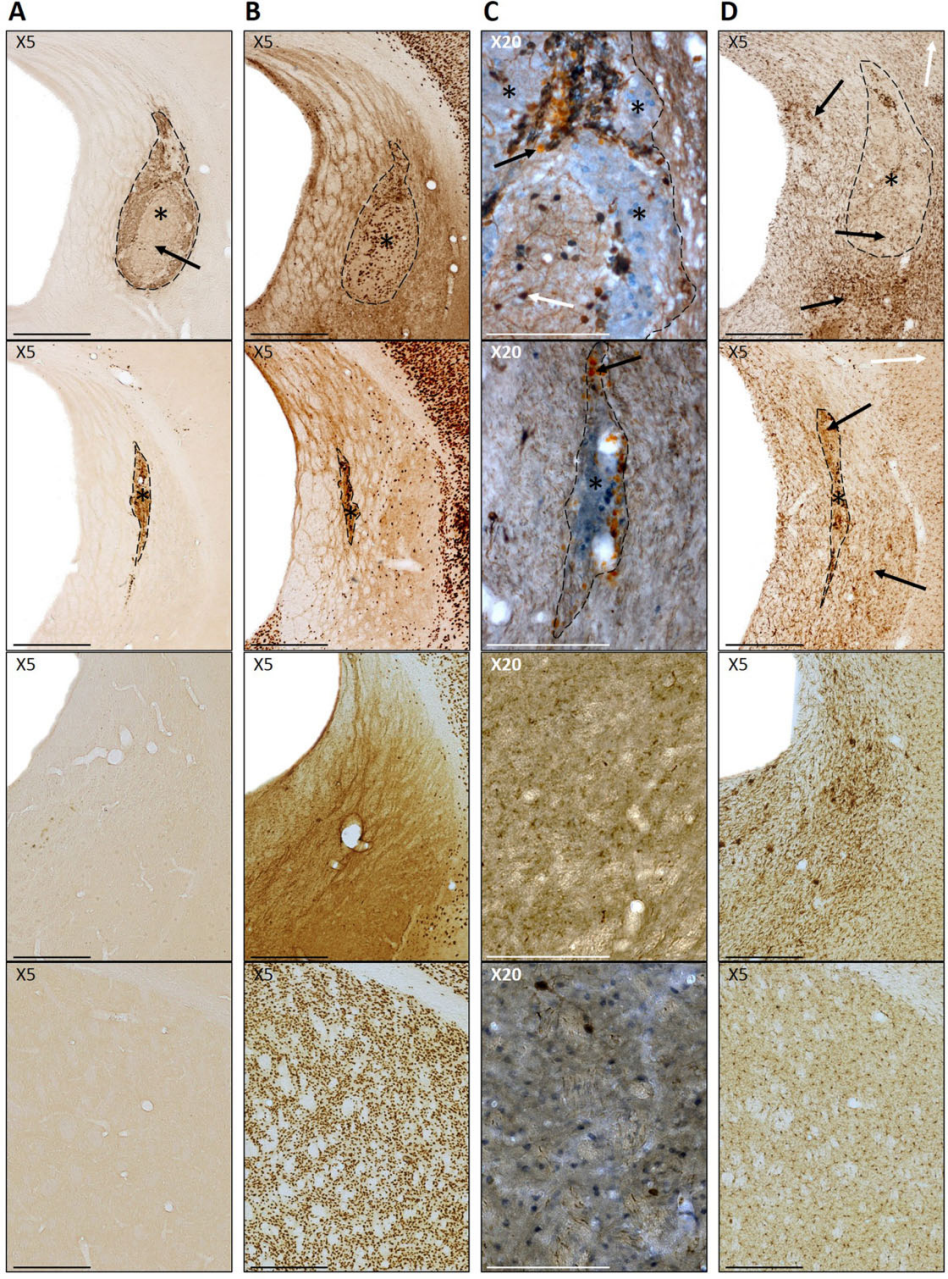
Photomicrographs of typical large and smaller grafts (first and second row, respectively), lesion only and control (third and final row, respectively). (A) GFP^+^ staining identifying Chrm4-EGFP-CD1-grafted tissue (indicated by *) within the host parenchyma. Paler areas of **Fig. 1.** (Continued) non-medium spiny neurons cell types are seen within the graft (indicated by arrow). Scale bar represents 500 μm. (B) NeuN^+^ staining of mature neurons. Areas of grafted cells can be clearly identified within the lesioned striatum (*). Scale bar represents 500 μm. (C) DARPP-32^+^ staining (blue) shows distinct P-zones within the grafts (*). Parvalbumin^+^ interneurons (brown stain; white arrow) are present throughout the grafts. Black arrows highlight the nonspecific orange-colored staining of spherical dead cells. Scale bar represents 100 μm. (D) Iba1^+^ staining of microglia. Resting-state ramified cells (white arrow) can be seen on the peripheral cortex areas. Clusters of darker, amoeboid activated cells (black arrow) can be seen within the grafts (*) and the surrounding striatum. Scale bar represents 500 μm.

The location of grafts in the C57BL6/J hosts was identified through immunohistochemical labeling of the transplanted Chrm4-EGFP-CD1 tissue using an anti-GFP antibody (A11122; Invitrogen, Loughborough, UK), and corresponded to clearly identifiable regions of NeuN^+^ staining within the lesioned striatum (Fig. 1A). CD1 hosts were transplanted with CD1 tissue, and therefore could not be identified through GFP staining, consequently NeuN^+^ staining was used to identify the graft location in these animals. The presence of fully differentiated adult neurons (NeuN^+^ cells) within the grafted area was used to determine graft survival in all groups (Fig. 1B), with grafts with no positive NeuN staining excluded from graft analyses. These animals were however retained in the analysis of microglial immune response. It is important to note that while successful grafts are defined here as those containing NeuN^+^ cells, survival of other cell types, such as immature neurons and glial cells, cannot be excluded. Volumes were calculated by measuring cross-sectional areas of NeuN^+^ (total graft volume) and DARPP-32^+^ graft regions (P-zones) across 1:12 series and using the formula: volume = (Σ*A* × *M*)/*f*, where *A* = area of graft (µm^3^), *M* = section thickness (µm), and *f* = section frequency (Fig. 1B and C).

For larger grafts, total cell numbers were calculated by unbiased stereology. For smaller grafts, stereological analysis would generate a large sampling error, therefore these were counted manually using Image J v1.45 software (National Institutes of Health (NIH), Bethesda, MD, USA) following imaging of grafted sections. Mean cell diameter was obtained for NeuN^+^, DARPP-32^+^, and parvalbumin^+^ cells by measuring the minimum and maximum diameters of 10 cells per graft using Image J.

Iba1-labeled series were used to grade the host microglial response in the grafted area using an established semi quantitative rating scale^23^. Each section was graded 0 to 4 according to the following categories: (0) no specific activated microglia in the graft area, (1) low number of activated microglia distributed as scattered single cells or clustered in a few small patches in or around the graft, (2) several activated microglia distributed as single cells or clustered in multiple prominent patches, (3) dense immunostaining of the graft area and a large number of activated microglia in and around the graft, and (4) very dense immunostaining of the whole graft area and a very large number of activated microglia in and around the graft. Activated microglia were easily identified by their morphological appearance^24^ (Fig. 1D). The highest grade given to any section for each animal was the grade assigned to that animal.

As TP were not dissociated, transplants were prepared by WGE units rather than by cell number as in the CS preparations. Embryos used for CS and TP were collected from the same litters, so although cell number could not be determined, an estimate of the number of cells per WGE at each age was calculated using total counts from the CS and dividing by the total number of WGEs dissociated (see Table 2). Since E12 WGE contained approximately half the number of cells of E14 WGE, a pair of E12 WGEs were transplanted for each E14 WGE to maintain a consistent total cell number, as close to 500,000 as possible. However, transplanting different proportions of WGE raises the issue that the E12 TP grafts of 2 WGEs may have twice the proliferative potential of the single WGE E14 TP. As it is not possible to control for both cell number and quantity of WGE transplanted, graft outcome measures were subsequently transformed to account for the proportion of WGE transplanted as described below:

Cell counts and volume data were corrected for the proportion of WGE transplanted using the following transformations: *Tn = n/*proportion of WGE transplanted and *T*vol *=* vol/proportion of WGE transplanted, where proportion of WGE transplanted = number of cells transplanted/mean number of cells in WGE, *Tn* = corrected cell count, *n* = actual cell count, *T*vol = corrected volume, and vol = actual volume.

Transformed data from successful grafts in all groups were analyzed together using 3-way analyses of variance (ANOVAs) in Genstat for Windows v16.1 software. If a significant main effect of strain was found, then B-S and W-S groups were subsequently analyzed in separate 2-way ANOVAs. Consequently, NeuN^+^ graft volume, DARPP 32^+^ graft volume, DARPP-32^+^ cell count, proportion of DARPP-32^+^ cells, parvalbumin^+^ cell counts, proportion of parvalbumin^+^ cells, and activated microglia scores were analyzed in separate ANOVAs for the B-S and W-S groups. For immune response data, transplanted mice with no detectable surviving grafts, as well as lesion only controls, were also included in the analyses.

## Results

Mouse donor cells from E14 and E12 WGE were prepared either as standard dissociated single-CS or as nontriturated partially digested TPs. These cell preparations were transplanted into the striatum of 2 commonly used laboratory mouse donor/host strain paradigms; a B-S and a W-S model. Grafts were analyzed using immunohistochemistry 12 wk later and graft size, neuronal content, DARPP-32^+^ MSN, and parvalbumin^+^ interneuron number compared, as well as the microglial reaction to the graft by the host. CS of each donor age was also analyzed after 24 h and 7 d *in vitro* to assess whether the age at which tissue is harvested affects the development of cells independently from the host environment.

### Graft Survival

The presence of DAB-labeled GFP^+^ Chrm4-EGFP-CD1 donor cells corresponded with areas of NeuN^+^ and DARPP-32^+^ staining in the C57BL6/J hosts, confirming the donor origin of the cells (Fig. 1A and B). Transplanted cells could be clearly identified within the lesioned host striatum by staining for NeuN^+^ mature neurons in all hosts, including those transplanted with non-GFP donor cells (Fig. 1B). The proportion of surviving grafts for each group and raw untransformed data for surviving grafts are shown in Table 1. There was no effect of donor/host on NeuN^+^ graft survival (*t*_6_ = 0.208, *ns*), and a high proportion of NeuN^+^ grafts was identified in all groups (80% to 100%) except for E14 TP, of which only 5 of 13 (43%) transplanted mice had NeuN^+^ cells in the grafted region after 12 wk. Graft volumes varied both within and between groups, ranging from just 12 × 10^6^ µm^3^ up to 588 × 10^6^ µm^3^.

### Graft Volume and Cellular Composition

**Fig. 2.**
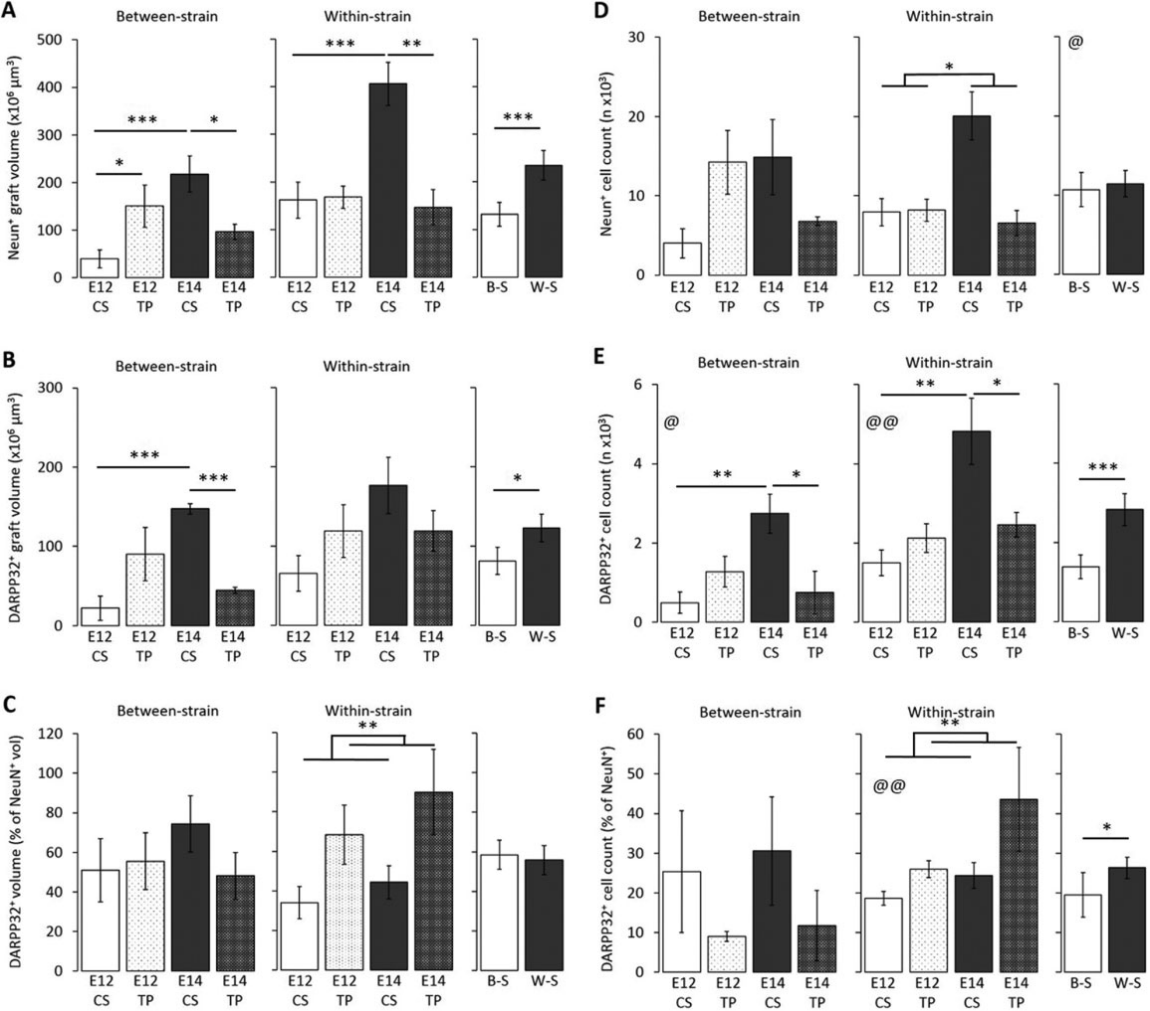
(A) NeuN^+^ graft volumes. Within-strain (W-S) transplants were larger than the between-strain (B-S) transplants (***P < 0.001). E14 tissue yielded a larger volume than E12 tissue when transplanted as single-cell preparation (CS) in both strain models (***P < 0.001). E14 tissue produced larger grafts when transplanted as CS than tissue piece (TP) style preparation in B-S (*P < 0.05) and W-S (**P < 0.01) models. E12 tissue yielded a larger volume when transplanted as TP than CS in the B-S model only (*P < 0.05). (B) DARPP-32^+^ graft volumes. W-S transplants contained a larger volume of DARPP-32^+^ tissue than the B-S (*P < 0.05). The B-S CS transplants yielded a larger DARPP-32^+^ volume using E14 tissue than E12 (***P < 0.001), and E14 tissue yielded a larger volume when transplanted as CS than TP (***P < 0.001). (C) Proportion of DARPP-32^+^ graft tissue (proportion = [DARPP-32^+^ volume/NeuN^+^ volume] × 100). There was no difference in the proportion of DARPP-32^+^ tissue in the different models; however, CS transplants yielded a higher proportion of DARPP-32^+^ tissue in the B-S groups than in the W-S groups (*P* < 0.05). TP yielded a higher proportion of DARPP-32^+^ tissue than CS in the W-S groups (**P < 0.01). (D) NeuN^+^ graft cell counts. No effect of model on neuronal cell counts was detected. E14 tissue yielded a greater number of neurons than E12 (@*P* < 0.05). CS transplants contained more NeuN^+^ cells than the TP in the W-S transplants (*P* < 0.05); however, TP grafts comprised of more NeuN^+^ cells than CS at age E12 (*P* < 0.05). (E) DARPP-32^+^ cell counts. More DARPP-32^+^ cells were present in the W-S grafts than the B-S (***P < 0.001). E14 tissue produced a greater number of DARPP-32^+^ cells compared to E12 in the B-S (@*P* < 0.05) and W-S model (@@*P* < 0.01). CS transplants yielded more DARPP-32^+^ cells using E14 tissue than E12 in both the BS (**P < 0.01) and W-S (**P < 0.01) models. E14 tissue yielded a higher DARPP-32^+^ cell count when transplanted as CS than TP in the B-S (*P < 0.05) and W-S (*P < 0.05). (F) DARPP-32^+^ cell counts as a proportion of total NeuN^+^ cells. W-S grafts yielded a greater proportion of DARPP-32^+^ cells within the graft than the B-S (*P < 0.05). In the W-S groups, E14 tissue produced a greater proportion of DARPP-32^+^ cells compared to E12 (@@*P* < 0.01) and TP preparations produced a greater proportion than CS (*P < 0.01).

Figure 2A shows the volumes of NeuN^+^ tissue in the surviving grafts for each group and a comparison of mean graft volume of B-S (Chrm4-EGFP-CD1 tissue into C57BL6/J hosts; B-S) and within strain (CD1 tissue into CD1 hosts; W-S) groups. Grafts from the W-S group were significantly larger than those observed in the B-S group (*F*_1, 27_ = 19.08, *P* < 0.001). Preparations of E14 CS yielded significantly larger grafts than E14 TP in the B-S model, while the younger E12 tissue produced larger grafts when prepared as TP than as CS (age × preparation: *F*_1, 11_ = 14.52, *P* < 0.01). In the W-S model, E14 CS also yielded significantly larger grafts than E14 TP; however, there was no significant difference between grafts derived from different preparations of E12 tissue (age × preparation: *F*_1, 16_ = 17.14, *P* < 0.001).

Distinct regions of DARPP 32^+^ staining were observed within all surviving grafts (Fig. 1C). The volume of DARPP-32^+^ patches (P-zones) within each graft is shown in Fig. 2B. W-S transplants yielded significantly larger total P-zone volumes than B-S (strain: *F*_1, 27_ = 6.50, *P* < 0.05). B-S transplants contained larger P-zone volumes when transplanted as CS than TP at E14, while the reverse was true for E12 tissue (age × preparation: *F*_1, 11_ = 18.27, *P* < 0.001). A similar trend was observed in the W-S groups; however, a statistically significant interaction was not found. As Fig. 2C shows, there were no differences in the proportion of DARPP-32^+^ P-zone volume (out of total NeuN^+^ graft volume) in B-S and W-S groups. B-S transplants showed a trend toward higher proportion of P-zones in E14 CS compared to E14 TP, although this was not statistically significant. No difference in the B-S E12 preparations was observed. W-S E12 groups again showed a tendency for higher proportions of P-zone tissue from E12 transplants as TP rather than CS. However, at E14, TP produced the larger DARPP-32^+^ proportion compared to CS—the only measure in which E14 TP outperformed E14 CS (strain × preparation: *F*_1, 27_ = 10.73, *P* < 0.01).

There was no difference between B-S and W-S transplants in the total number of mature NeuN^+^ neurons within the grafts (Fig. 2D). Cell counts reflected the data patterns observed in graft volume, with E14 CS yielding more cells than E14 TP, and E12 TP yielding more cells than E12 CS (age × preparation: *F*_1, 27_ = 23.43, *P* < 0.001). The W-S grafts contained more DARPP-32^+^ cells than B-S (strain: *F*_1, 27_ = 21.43, *P* < 0.001; Fig. 2E). Grafts of E14 tissue contained more DARPP-32^+^ cells than those of E12 origin in both W-S and B-S groups (age: *F*_1, 16_ = 13.6, *P* < 0.01 and *F*_1, 11_ = 6.71, *P* < 0.05, respectively). E14 tissue yielded higher DARPP-32^+^ content when transplanted as CS than TP, while there was trend for E12 to produce more as TP in both W-S and B-S groups (age × preparation: *F*_1, 16_ = 8.94, *P* < 0.01 and *F*_1, 11_ = 17.25, *P* < 0.01, respectively).

The W-S models yielded the greatest proportion of DARPPP-32^+^ cells within the grafts compared to the B-S group (strain: *F*_1, 27_ = 4.46, *P* < 0.05; Fig. 2F). Grafts derived from E14 tissue contained a higher proportion of DARPP-32^+^ cells than those from E12 in the W-S model (age: *F*_1, 16_ = 9.88, *P* < 0.01). In addition, TP preparations in the W-S model yielded a greater proportion than the CS (preparation: *F*_1, 16_ = 12.97, *P* < 0.01).

**Fig. 3.**
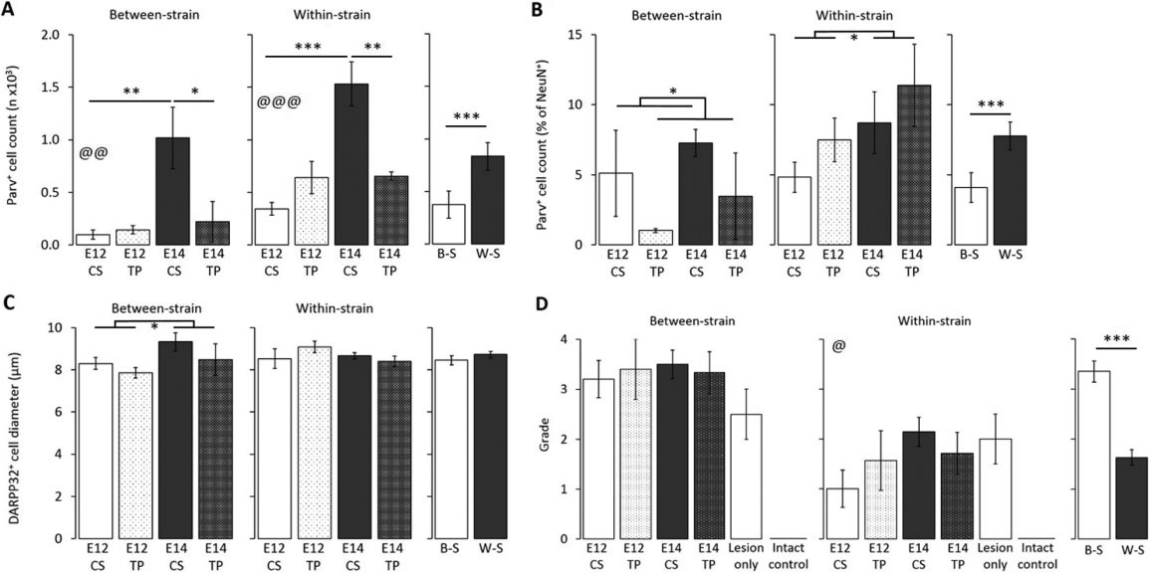
(A) Parvalbumin^+^ cell counts. A greater number of parvalbumin^+^ interneurons were present in the within-strain (W-S) model than the between-strain (B-S) model (***P < 0.001). E14 tissue yielded a greater number of parvalbumin^+^ interneurons than E12 in both the B-S (@@*P* < 0.01) and W-S (@@@*P* < 0.001) models. E14 tissue yielded a higher parvalbumin^+^ cell count when transplanted as single-cell preparation (CS) than tissue piece style preparation (TP) in the B-S (*P < 0.05) and W-S (**P < 0.01) models. All data presented in Fig. 1 are adjusted for proportion of whole ganglionic eminence transplanted. (B) Parvalbumin^+^ cell counts as a proportion of total NeuN^+^ cells. W-S grafts yielded a greater proportion of parvalbumin^+^ cells within the graft than the B-S (***P < 0.001). E14 tissue produced a greater number of parvalbumin^+^ cells compared to E12 in the W-S (*P < 0.05). CS preparations produced a greater number of parvalbumin^+^ cells compared to TP in the B-S (*P < 0.05). (C) DARPP-32^+^ cell diameter. There was no difference in DARPP-32^+^ cell diameter between the B-S and W-S groups. Cells derived from E14 tissue were larger than those taken from E12 tissue in the B-S groups (*P < 0.05). (D) Grading score for activated microglia (0 to 4) in the grafted striatum in the B-S groups, W-S groups, and the mean grading score for activated microglia in all grafted groups. Higher levels of activated microglia were found in B-S transplants than W-S (***P < 0.001). W-S transplants of E14 tissue produced an increased microglial response than E12 (@*P* < 0.05). No effect of age on microglial response was found in the B-S groups.

Significantly more parvalbumin^+^ cells were found in W-S transplants compared to B-S (strain: *F*_1, 27_ = 20.67, *P* < 0.001; Fig. 3A). In addition, E14 generated more parvalbumin^+^ cells than E12 tissue in both W-S and B-S groups (age: *F*_1, 16_ = 19.39, *P* < 0.001 and *F*_1, 11_ = 14.14, *P* < 0.01, respectively), and CS yielded more than TP preparations (preparation: *F*_1, 16_ = 4.49, *P* < 0.05 and *F*_1, 11_ = 8.05, *P* < 0.05, respectively) although this effect was mostly due to very high numbers in the E14 CS groups compared to all other combinations. E14 CS grafts contained significantly more parvalbumin^+^ cells than E14 TP in both W-S and B-S groups (age × preparation: *F*_1, 16_ = 18.40, *P* < 0.001 and *F*_1, 11_ = 10.13, *P* < 0.01, respectively), and there was a trend for E12 TP to yield more than E12 CS, but this did not reach significance.

The proportion of parvalbumin^+^ cells (as a percentage of NeuN^+^ cells) was greatest in the W-S model (strain: *F*_1, 27_ = 12.97, *P* < 0.001; Fig. 3B). CS preparations yielded a higher proportion compared to TP in the B-S groups (Preparation: *F*_1, 11_ = 7.13, *P* < 0.05), and E14 tissue yielded a higher proportion than the E12 in the W-S groups (age: *F*_1, 16_ = 5.76, *P* < 0.05).

There was no difference in the diameter of the DARPP-32^+^ cells between the B-S and W-S groups; however, those derived from E14 tissue in the B-S groups were significantly larger compared to those from E12 tissue (age: *F*_1, 13_ = 12.98, *P* < 0.01).

### Microglial Response

Iba1 labeling revealed dense areas of microglial activation, not only within the grafted area but also extending beyond the transplant boundaries to the host striatum in all mice except for intact control animals (Fig. 1D). Numerous dead cells and cellular debris were observed within most grafts and needle tracts, visible in sections stained with DAB as spherical clusters of paler staining (arrows in Fig. 1C).

Figure 3D shows the graded microglial response for each group. Activation of microglia was significantly higher in B-S than in W-S groups (*F*_1, 39_ = 99.09, *P* < 0.001). Grafts derived from E14 tissue in the W-S model induced a greater microglial activation than those of E12 tissue (*F*_1, 23_ = 5.54, *P* < 0.05); however, this effect was not seen in the B-S groups. No difference was detected between TP and CS preparations.

### Differentiation In Vitro

**Fig. 4.**
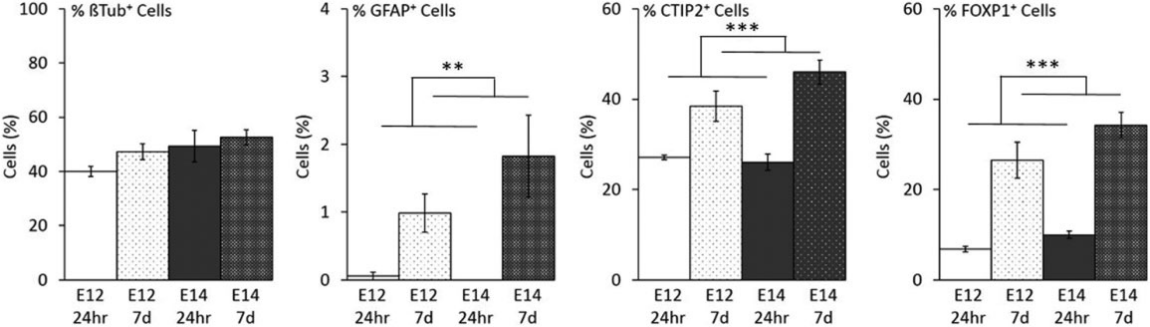
Cell counts from plate downs of 100,000 cells from E12 and E14 single-cell suspensions after 24 h and 7 DIV. No differences were observed in the number of neurons across groups (βTub^+^ staining). Compared to 1 DIV, the cultures at 7 DIV contained a significantly higher percentage of astrocytes (**GFAP^+^, *P* < 0.01) and medium spiny neurons precursors (***CTIP2^+^, *P* < 0.001; ***FOXP1^+^, *P* < 0.001).

To investigate the development and maturation of cells from E12 and E14 donor embryos independent of the host environment, CS from CD1 embryos was prepared as described for transplantation and cultured for 24 h and 7 d *in vitro*. As TP preparations were not dissociated, it was not possible to culture these comparably. Cell counts from primary cultures are shown in Fig. 4. There was no difference in the proportion of β-tubulin^+^ cells across any age or time point; however, there was a trend toward a greater number of β-tubulin^+^ cells at 7-d post plate-down compared to 24 h, as well as for E14 compared to E12. Very few GFAP^+^ cells were found in any group; however, there were significantly more after 7 d in both E12 and E14 donor age groups (*F*_1, 8_ = 16.99, *P* < 0.01). There was a significant increase in the proportion of CTIP2^+^ cells at 7 days *in vitro* (DIV) compared to 24 h (*F*_1, 8_ = 44.52, *P* < 0.001) but there was no effect of embryonic age. FoxP1^+^ MSN precursor cells also accounted for a higher proportion of the population at 7 DIV compared to 24 h (*F*_1, 8_ = 78.21, *P* < 0.001) with no effect of embryonic age.

## Discussion

The effect of donor age, cell preparation (single CS or dissociated TP), and variability in donor/host strain on primary embryonic striatal graft development and the host microglial response was investigated. Preparation as a CS is the method most routinely used, with trituration following enzymatic digestion to form a quasi-single CS. The TP style preparation used in this study, although not an identical treatment to other chopped tissue piece preparations,^25–27^ provides a less severe treatment than standard CS protocol^28,29^. Cells underwent the same enzymatic digestion to aid in the transplantation process, but did not undergo manual trituration, thus leaving the tissue relatively intact, thus theoretically reducing cell stress. To provide information on how model selection could affect the host response to transplants and subsequent graft survival/development, 2 different donor/host strain combinations were used: a B-S model transplanting Chrm4-EGFP-CD1 tissue into C57BL6/J hosts and a W-S model with CD1 tissue transplanted into CD1 mice.

A high percentage of graft survival was found across all groups, except for E14 TP in both strains, which was the least effective transplant protocol in terms of graft survival (see Table 1). These data show that transplanted cells can survive under a variety of protocol conditions, yet survival rates were still not as high as usually seen in rat studies, and considerable variation in graft volume and content was seen within experimental groups. Graft cells were analyzed for the expression of the mature neuron marker NeuN, MSN marker DARPP-32, and the interneuron marker parvalbumin. Some NeuN^+^ cells did not appear to express either DARPP-32 or parvalbumin and could be MSN cells not yet producing DARPP-32, nonstriatal neural cells, or nonparvalbumin interneurons.

### Donor Age

In general, E14 tissue produced grafts containing a higher number of mature neurons, DARPP32^+^ MSN cells, and parvalbumin^+^ interneurons compared to preparations transplanted using E12 tissue after considering the number of progenitor cells transplanted. Neural graft volume was larger for E14 preparations than E12 in the W-S groups, and this trend was also seen in the B-S groups, although not reaching significance—possibly due to small group sizes.

CS preparations produced more NeuN^+^ cells, larger graft volumes, and more DARPP32^+^ and interneuron content when harvested at E14 than at E12. Additionally, B-S transplants of E14 CS produced a higher proportion of P-zone tissue than E12 CS. In contrast, there was no effect of age on TP in any of the above measures, although a consistent trend was apparent showing the opposite effect, with TP yielding better grafts at E12 than at E14. Striatal transplants of E14 preparations in rats have been shown to produce larger grafts and DARPP-32^+^ P-zones within the graft compared to older tissue as well as the greatest functional recovery.^10,30,31^ Given that the developmental stage at E14 in rats is equivalent to age E12.5 in mice, by comparing the Carnegie stages of development^32^, it would be expected that E12 TP in mice should reflect the results seen in E14 TP rat studies. It is possible that the digestion process and trituration of the mouse CS have more of a detrimental effect on the cells at this younger age than at E14 and are less tolerant to the treatment than rat cells. This could lead to a reduced capability of mouse E12 CS cells to survive and develop posttransplantation. In addition, it is possible that the Carnegie stages are not perfectly translated from rat to mouse and E12 could be more representative of a younger stage than the estimated E14 rat stage. This could have important implications for fetal age selection in primary human tissue transplants.

No effect of donor age was found in the *in vitro* measures investigated, including numbers of mature neurons (β-tubulin), early MSNs (FoxP1, CTIP2), and astrocytes (GFAP). E12 and E14 cells were equally viable at the time of transplantation/cell plating. However, *in vitro* conditions are not reflective of the *in vivo* environment, and differences seen *in vivo* may suggest that it is the interaction of cells with the host environment affecting the apparent differences in development. It has been shown that neuronal cells under stress are more likely to be destroyed by the host^33^, therefore if younger cells are more susceptible to stress, they may be more susceptible to the host immune response. The high levels of activated microglia seen within the grafted regions, and even in the lesion only controls, confirm that the immune response could play a critical role in the long-term survival of cells^14^.

Parvalbumin^+^ interneurons were more abundant in grafts derived from E14 CS than those from any other, an indication that these grafts may contain a greater proportion of this interneuron population than the other groups, thereby presenting a cell population more characteristic of the normal striatum^34^. To obtain the neural diversity in grafts closest to that seen in the adult striatum, it is necessary to transplant both the lateral ganglionic eminence (LGE) and the medial ganglionic eminence (MGE)^13,35^. In mice, the LGE, the source of striatal progenitors^36^, is visible by E12, while the MGE, where interneurons are born, is visible as early as E11^35,37^, indicating that E12 might be the earliest time point for obtaining all the necessary cell types in mice. It is known that interneuron populations contribute to normal striatal function and development^35^, and these may be playing a supportive role in the development of the MSNs within the graft^38^. The MGE is much larger at E14 than at E12, and as this is the origin of interneuron progenitors^39,40^, would most likely contribute a greater proportion of interneurons to the transplanted population. In turn, this may have resulted in the improved development of E14 grafts^13,35^.

It is interesting to note that the mean cell body diameter of DARPP32^+^ grafted cells from Chrm4-EGFP-CD1 tissue was significantly larger in the E14 age groups than in the E12, although this was not seen in the CD1 grafts. Given that at the time of perfusion all grafted cells were 12 wk old, under normal physiological conditions, it would be expected that they would have reached the same level of maturity and hence size. This may be an indication that the E14 cells are less inhibited by the local environment after transplantation into the host than the E12 cells, although contrasting evidence suggests that older cells, once past their proliferative stage, may be able to compensate less well^12^. Alternatively, E12 tissue could be undergoing proliferation for a longer time posttransplantation, thus giving rise to cells until much later. These, at the time of sacrifice, could be less mature than those born closer to the time of transplantation. Changes in cell size may also be because of shrinkage or swelling due to physiological processes. For example, increases in cell size have been linked to necrosis^41^, while cells in the early process of apoptosis are reduced in size^42^. In this case, it is possible that more E14-derived cells could be necrotic or that more E12-dervived cells are undergoing apoptosis; however, this could not be determined within the current study. No difference in the cell size of parvalbumin^+^ interneurons was seen (data not presented).

### Cell Preparation

There are potential benefits of delivering the transplant as tissue pieces rather than triturated CSs. Limiting the manipulation of the tissue can reduce disruption and death of neuronal populations within the preparation, and the retention of the extracellular matrix may protect cells during transplantation and in the initial postgraft period. However, it has been suggested that the transplantation of whole tissue pieces may induce a stronger immune response due to the presence of the intact donor vasculature and antigen presenting cells (APCs)^26,43^. Although the use of nonimmunogenic bioengineered scaffolds could avoid this issue^44,45^, protocols generally require dissociation of cells prior to seeding into a scaffold, therefore still posing a risk to neuronal populations. Preparing tissue as partly digested tissue pieces without trituration^28,29^ may prevent disruption to MSN precursors prior to transplantation and thus improve graft survival.

The results show a significant difference in the effect of preparation type on graft morphology depending on the age of the tissue used. E14 tissue prepared as CS produced grafts that are phenotypically superior to those transplanted as TP in almost all parameters including graft survival. Dissociated cell preparations are thought to provoke less of a host immune response when transplanted because the immunogenic donor vasculature is at least partially destroyed prior to implantation^43,46^. In addition, trypsinized single-cell preparations provide an advantage over solid pieces of tissue by potentially allowing transplanted cells access to the host capillary network more easily. The necessity of establishing contact in order to nourish the grafted tissue was demonstrated early on in studies implanting in vessel-rich and vessel-poor microenvironments^47^. Rat-to-rat grafts from CS transplants produce a greater proportion of striatal-like tissue, with more DARPP-32 expressing cell populations than those from TP, as well as providing greater innervation of the host parenchyma^29^. Cells transplanted as TP within a surrounding matrix may be restricted in terms of migration and integration into the host brain. The present study suggests that the benefits of transplanting dissociated CSs may outweigh those of a supportive matrix provided by TP transplants and that the trituration process is not too harsh to affect survival of the transplant at E14.

Conversely, E12 tissue produced larger grafts with greater striatal-like content when prepared as TP over CS. Previous studies in rats have shown that, for transplants of TP, older donor tissue is tolerated less and that younger tissue has a better chance of survival^11,12,47^ corresponding to what we find in mouse TP transplants. It is unclear why the dissociation processes involved in CS preparation would reverse this trend, although, as discussed above, it seems that mouse WGE tissue is better able to withstand dissociation when processed at E14 than at E12 as evidenced through E14 CS transplants yielding improved long-term graft survival and larger grafts. Studies have suggested that different subpopulations of rat neurons are more sensitive to trypsinization than others^12^. Mouse cells may also be more sensitive, particularly at different developmental stages, warranting a systematic study of the effect of trypsinization on mouse precursors.

E12 TP survived transplantation with an improved capacity to produce successful grafts, although it is unclear why the same results are not reflected with E14 TP. Potentially, the less mature cells within the E12 TP are more proliferative and migratory at this early stage of development, therefore not restricted by the surrounding matrix. The particularly low survival rate in E14 TP preparations may indicate that TP at this age are not as amenable to integration as those at E12, potentially due to an increased potential of their vasculature and APCs to induce an immune response in the host^43^. In addition, following expulsion from the graft cannula, cells within the E14 TP might be more densely packed within the host striatum than single cells which could impede diffusion and timely integration with the capillary^18,46^.

### Strain Effects

The models selected for the purposes of this study were the 2 most commonly used paradigms within the lab, with the aim of determining how the choice of these particular models could affect the graft outcome.

We showed that the use of the different W-S and B-S models did not affect the number of surviving grafts. However, the transplants in the W-S model yielded the largest grafts in terms of neuronal volume compared to the B-S paradigm and had a higher number and proportion of DARPP32^+^ cells. The CD1 grafts also contained more interneuron cells. A previous study using the same Chrm4-EGFP-CD1 donor tissue observed much larger grafts and survival^19^, although notably this CD1-derived tissue was transplanted into CD1 hosts rather than the C57BL/6.

The results suggest that the choice of strain and matching of donor and host animals for transplantation studies could be critical in achieving robust results. Iba1 staining revealed a significant amount of microglial activation within the grafted areas of all mice except the intact controls, including those in the lesion only group and those with no detectable surviving grafts. A significantly higher grading of activated microglia was found in the B-S than in the W-S groups. Allotransplants elicit a greater immune response than isogenic tissue, and while neither of the models investigated here are inbred strains, it is clear that the response is increased when immunological disparity is greater^43^. This, in turn, can be linked to reduced transplant survival. Since the CD1 hosts received tissue derived from the same strain, it is likely that this was tolerated more than the tissue in the mismatched B-S groups. In addition, some studies have shown that the GFP marker associated with the Chrm4-EGFP-CD1 donor tissue could in itself be immunogenic^48^, although it is unclear if this is the case in striatal transplants in the C57BL/6 model^49^. It is also plausible that the C57BL/6 strain is inherently more prone to an exaggerated inflammatory response compared to the CD1 mice. It has been demonstrated that C57BL/6 mice have a strong bias to M1 inflammatory reaction, whereas other strains, such as Balb/c, tend toward a more supportive M2 response^50^. The separation of the effects of immunogenicity of the different donor tissues used and the reactivity of the hosts is beyond the scope of this study; however, it does warrant further investigation.

E14 tissue transplanted into the W-S models induced a greater microglial reaction than the E12 tissue. It is possible that tissue pieces transplanted from later embryonic ages contain more vasculature and hence could invoke a greater immune response, although this is yet to be tested. The fact that this pattern was not detected in the B-S model could be a result of a ceiling effect since the microglial response was consistently high in all B-S groups.

The higher levels of activated microglia in the C57BL6/J hosts could explain the lower surviving cell number and graft volume^51,52^. It was noted that the area of activation exceeded the area of transplantation, suggesting secondary activation or recruitment of microglia to the site of transplantation. Interestingly, the glial response appeared reduced in individuals with rejected grafts, presumably because the transplanted cells had already been subjugated and the immune response had entered a post reactive phase. The ongoing proliferation of activated glial cells in and around the grafts is suggestive of ongoing reactivity with the surviving implanted cells. This could be an indication that the grafts surviving to 12 wk may be hampered long-term by the immune response of the hosts. Therefore, the study of immunosuppressive regimes in mouse to mouse transplantation could be a key to resolving the less than optimum quality of the grafts seen, as immunosuppression is generally only considered to be required for xenotransplant models.

## Conclusions

The results highlight a capacity of mouse transplants to survive under a variety of conditions and a need for protocols to be optimized to improve consistency and reliability. Donor age and tissue preparation technique are important factors that affect the morphology of primary fetal grafts. The data from this study suggest that more successful grafts are derived from single-cell preparations of E14 tissue or from less dissociated tissue pieces at E12.

We found large variation in grafts across all the experimental groups, which implies the influence of other factors that may be more fundamental than the methodological modifications investigated in this study. Any impact of changes in cell preparation or donor age may be reduced by other more influential factors in the mouse to mouse model, highlighted by the differences between the strains investigated here. High levels of activated microglia in the grafted zones, particularly in the B-S transplants, and the presence of dead cells in all groups suggest that further investigation into immune response of mouse hosts to specific tissues is warranted.
